# Possible involvement of the RARRES2/CMKLR1-system in metabolic and reproductive parameters in Holstein dairy cows

**DOI:** 10.1186/s12958-019-0467-x

**Published:** 2019-02-18

**Authors:** Namya Mellouk, Christelle Ramé, Mélodie Diot, Eric Briant, Jean-Luc Touzé, Daniel Guillaume, Pascal Froment, Joëlle Dupont

**Affiliations:** 1 INRA UMR85 Physiologie de la Reproduction et des Comportements, F-37380 Nouzilly, France; 2 France CNRS UMR7247 Physiologie de la Reproduction et des Comportements, F-37380 Nouzilly, France; 3 France Université François Rabelais de Tours F-37041 Tours, France IFCE, F-37380 Nouzilly, France; 4 INRA - Unité Expérimentale du Pôle de Physiologie Animale de l’Orfrasière de Tours UEPAO 1297, F-37380 Nouzilly, France; 5 Unité de Physiologie de la Reproduction et des Comportements, Institut National de la Recherche Agronomique, 37380 Nouzilly, France

**Keywords:** Adipose tissue, RARRES2, CMKLR1, Energy content, Granulosa cells, Lactating cows

## Abstract

**Background:**

In dairy cows, the energy cost of milk yield results in a negative energy balance (EB) and body fat mobilization that impairs reproductive efficiency. Emerging evidence suggests that the novel adipokines, Retinoic acid receptor responder protein 2 (RARRES2), and its main receptor, Chemokine-like receptor 1 (CMKLR1) are involved in the regulation of metabolic and ovarian functions. So, we investigated in a first experiment the plasma RARRES2, and RARRES2 and CMKLR1 mRNA expression levels in subcutaneous adipose tissue (SAT) and granulosa cells (GC) at different times of body fat mobilization in dairy cows (4, 8, 20 and 44 weeks postpartum, wk. pp. for SAT and 8, 20 and 44 wk. pp. for GC). Then, in a second experiment we examined the effect of high (HE) and low energy (LE) diets on the RARRES2 system and its links with metabolic and reproductive parameters.

**Methods:**

The first experiment included 9 animals fed with HE diet from 4 to 44 wk. pp. and the second one included animals fed either a HE diet (*n* = 8) or a LE diet (*n* = 8) from − 4 to 16 wk. peripartum. In both experiments, various metabolic and reproductive parameters were determined and associated with plasma RARRES2 as measured by bovine ELISA. RARRES2 and CMKLR1 mRNA expression levels were analyzed by RT-qPCR in SAT after biopsy and GC after aspiration of follicles.

**Results:**

Plasma RARRES2 levels were higher at 4 wk. pp. as compared to 20 and 44 wk. pp. and they were positively correlated with body fat mobilization and milk yield. RARRES2 and CMKLR1 mRNA expression levels increased from 4 to 8 wk. pp. (fat mobilization, EB < 0) and remained unchanged at 20 and 44 wk. pp. (fat reconstitution, EB > 0) as compared to 4 wk. pp. in SAT. RARRES2 and CMKLR1 mRNA levels decreased from 8 to 44 wk. pp. in GC from small follicles. In the second experiment, plasma RARRES2 increased from − 4 to 8 wk. peripartum similarly in both LE and HE cows. In addition, the area under of plasma RARRES2 curve was highly negatively associated with the number of small follicles obtained in HE animals during the cycle before the first artificial insemination. In SAT of HE cows, RARRES2 mRNA expression decreased at 1 wk. pp. compared to − 4 and 16 wk. peripartum whereas opposite expression patterns were obtained for CMKLR1. Similar results were observed for CMKLR1 mRNA expression in LE cows while there was no variation in RARRES2 mRNA expression. Moreover, RARRES2 mRNA was higher expressed in LE than in HE cows at 1 wk. pp.

**Conclusions:**

The lactation-induced fat and energy mobilization influenced plasma RARRES2 profile and mRNA expression pattern of RARRES2 and CMKLR1 similarly in both SAT and GC. In addition, the energy content of the diet did not affect plasma RARRES2 but it altered RARRES2 mRNA expression in SAT and the area under the curve of plasma RARRES2 that was negatively associated to the number of small follicles in HE animals. Thus, RARRES2 could be a metabolic or ovarian signal involved in the interactions between metabolic and reproductive functions in dairy cows.

**Electronic supplementary material:**

The online version of this article (10.1186/s12958-019-0467-x) contains supplementary material, which is available to authorized users.

## Background

Emerging evidence indicates that bioactive molecules produced by white adipose tissue, named adipokines are not only involved in energy metabolism but also in the regulation of reproduction and more precisely in the ovarian functions [1, 2]. Retinoic acid receptor responder protein 2 (RARRES2) is an adipokine expressed in various tissues, mainly in white adipose tissue and liver in mammals [3, 4]. It is produced as an inactive protein, which requires a successive proteolytic cleavage at its C-terminus to get an active form [5]. To mediate its physiological functions, RARRES2 is able to bind predominantly to a G protein-coupled receptor, the chemokine-like receptor 1 (CMKLR1) [6, 7]. Increased serum concentrations of RARRES2 have been reported in obese humans as well as in preclinical rodent models of adiposity [8]. In humans, RARRES2 plasma concentrations decrease after bariatric surgery and are associated with body mass index [9]. Previous studies in mice and humans indicate that RARRES2 regulates peripheral insulin signalling, glucose homeostasis, lipid metabolism and adipogenesis through autocrine and paracrine mechanisms [1, 7, 10, 11]. Lack of RARRES2 or CMLKR1 expression in vitro abrogates adipogenesis [1]. Recent studies showed that RARRES2 and CMKLR1 are expressed in the reproductive tract and could regulate gonadal functions [12]. Recombinant RARRES2 was found to suppress both basal estradiol secretion in granulosa cells (GC) from normal rats and FSH-induced progesterone and estradiol secretion in cultured preantral follicles and GC in vitro, suggesting the possible involvement of RARRES2 in the regulation of ovarian follicle growth and function [13, 14].

In bovine species, Song et al. [3] have cloned the full-length cDNA sequence of RARRES2 and CMKLR1 from adipose tissue. The sequence homology is species dependent: humans (79% (RARRES2) and 84% (CMKLR1)), mice (69% (RARRES2) and 79% (CMKLR1)), and pigs (86% (RARRES2) and 88% (CMKLR1)) [3]. Since this discovery, other investigators found single nucleotide polymorphisms (SNP) in the bovine RARRES2 encoding gene that are associated with carcass traits [15, 16]. Bovine RARRES2 transcript expression in subcutaneous adipose tissue (SAT) was associated with body condition score, yearling weight, body fat mobilization and adipocytes differentiation [3, 17, 18, 19].

After weaning, the expression of RARRES2 mRNA is increased in adipose tissue while it is unchanged in liver of calves [20]. In vitro treatment with nicotinic acid increases the expression of RARRES2 mRNA in bovine adipocytes and therefore might improve insulin sensitivity and/or adipocyte metabolism in dairy cows [21]. Moreover, we recently demonstrated that RARRES2 and CMKLR1 were expressed in bovine ovarian cells and RARRES2 decreased steroidogenesis and cholesterol production in response to IGF1 or FSH through CMKLR1 in GC and arrested oocyte maturation [22].

In dairy cows, the energy cost of milk production during early lactation leads to negative energy balance (NEB) and mobilization of body tissue reserves. The extent and duration of NEB may lead to some reproductive dysfunctions such as a reduced pulsatile LH release [23], and ovulatory failures [24]. These events are associated with modifications in adipokines (resistin, adiponectin and leptin) blood/serum or plasma concentrations, which are regulated by the energy level of the diet in dairy cows [25].

Since receptors of these adipokines are expressed in the reproductive tissues, our hypothesis is that these adipokines could convey body metabolic status to the reproductive tract to regulate fertility. However, the plasma profile of RARRES2 has never been investigated during early lactation and the late lactation period in dairy cows, yet it could be a key factor linking regulation of metabolism and reproduction in dairy cows.

Thus, our study aimed to determine firstly the plasma profile of RARRES2 at different times of body fat mobilization (4, 8, 20 and 44 wk. postpartum, pp) and to investigate the expression of RARRES2 and its main receptor, CMKLR1, in both SAT after biopsy and GC after aspiration of follicles. Then, in a second experiment we examined a potential effect of nutrition on the regulation of the RARRES2 system and its association with metabolic and reproductive parameters by analysing the influence of a high (HE) and a low energy (LE) diet.

## Methods

### Ethical issues

An ethics committee (Comité d’Ethique en Expérimentation Animale Val de Loire, CEEA VdL, protocol reference number 2012-10-4 and 01607.02) approved all experimental protocols, which were consistent with the guidelines provided by the French National Guidelines for Animal Care.

### Animals

The two independent experiments took place at the experimental unit UEPAO (Institut National de la Recherche Agronomique, Nouzilly, France). Animals were managed in a straw-bedded yard and the diet was distributed twice daily, at 0900 and 1500 h, and each cow had access to several feeders according to their diet (Insentec B.V., Marknesse, the Netherlands).

#### Experiment 1

Nine multiparous Holstein cows were fed with a HE diet from calving to 44 wk. pp. GC from aspiration of follicles, blood and SAT sampling were collected the same day. The animals were not inseminated.

#### Experiment 2

Thirty-nine primiparous Holstein dairy cows with similar body weight and back fat thickness were distributed in two dietary treatments groups. Animals were fed either with a HE diet calculated to yield 35 kg of milk/cow per day (*n* = 17) or a LE diet calculated to yield 25 kg of milk/cow per day (*n* = 22). The diets started from 4 wk. before the presumed first calving date to 16 wk. pp. The composition of the HE and LE diets was previously described [25] (Additional file 1: Table S1). The energy content of the diet was about 1.16-fold higher in the HE than in the LE diet (84.23 vs. 72.35 Mcal/kg of dry matter). Animals were artificially inseminated from 55 to 60 d pp. after spontaneous ovulation (no hormones used) 12 h after estrus if they had been detected by standing estrus or if the score obtained during 15 min of visual detection exceed 100 with the scoring scale established by Van Eerdendurg [26]. For the insemination, the semen of the same bull was used. For this present study we randomly chose eight animals by group, (HE: *n* = 8, LE: *n* = 8).

### Measurement of live body weight, variation in empty body weight, milk yield and dry matter intake

All procedures for the measurement of zootechnical parameters (live body weight, variation of empty body weight, milk yield, dry matter intake and back fat thickness) were conducted as previously described [25]. Briefly, after calving, cows were milked twice daily and weighed automatically after each milking (software RIC version RW1.7). As live body weight is affected by digestive contents, the estimation of empty body weight was corrected for digestive tract contents. A change of 4.5 kg of digestive contents per kg of dry matter intake was assumed [26]. Variation of empty body weight was calculated every day: empty body weight of the previous day was taken as the reference weight [27]. Dry matter intake was determined from the intake of fresh matter and the dry matter content of each feed of the ration. Concerning the analysis of the dry matter content of the ration and the feed compounds, a sample of corn silage was taken once a week and samples of concentrate and other feeds were taken in each new delivery (about once per month). Energy balance (Mcal/d) was calculated during the lactating period only as it was not possible to measure dry matter intake during the late lactation period according to the INRA feeding system (INRA, 2007). Adipose tissue mobilization was assessed through subcutaneous fat thickness measurements in the sacral region using ultrasonographic examination with a linear probe (LA 332 3.5/10.0-MHz transducer; Mylab30vet; R-Esaote, Hospimedi, Saint-Crépin-Ibouvillers, France), as previously described [28]. Back fat thickness was determined at 4, 8, 20 and 44 wk. pp. for Experiment 1 and at − 4, 1, 4, 8, and 16 wk. peripartum for Experiment 2 [29].

### Determination of reproductive parameters (experiment 2)

During the cycle before first artificial insemination, ovarian follicular dynamic of primiparous cows was monitored three times a week with a transrectal ultrasonographic examination using a linear probe (LV 513 6.0/8.0-MHz transducer; Mylab30; Esaote) allowing detection and measurement of antral follicles. Ovarian follicles were classified according to their diameter: small (SF, 3–5 mm), medium (MF, 5–7 mm) and large (LF, > 7 mm). The mean number of follicles in each class was calculated from the total number of follicles (SF, MF and LF) from the two ovaries per female divided by the number of ultrasonographic examinations. For the largest follicle, the precise diameter was noted. Based on the analysis of the ovarian scannings, we could follow the growth of each follicle and determine the number of follicular waves. Sirois & Fortune [30] have characterized a follicular wave by the development of one large follicle (dominant) and a variable number of smaller (non-dominant) follicles. The length of the cycle was determined combining ovarian scanning and estrus visual detection data. The beginning of the cycle was defined as the day of estrus detection (confirmed by ovulation). In case of silent ovulation, the beginning of the cycle was defined as the day when the dominant follicle of the ovulatory wave reached its maximal size before ovulation of the follicle was detected. The end of the cycle was the day of the first artificial insemination determined by estrus detection. Postpartum commencement of luteal activity (CLA) was defined as the first day when plasma progesterone concentration was higher than 0.70 ng/mL. This threshold was defined in order to 95% of the minima of points flanking the supposed day of estrus were above this threshold. The plasma progesterone concentration was considered as “negative” if lower than 0.70 ng/mL, “low” if between 0.70 and 3.80 ng/mL and “normal” if higher than 3.80 ng/mL. Pregnancy rate was determined at 35 d by ultrasonographic examination and at 90 d by transrectal palpation. It was defined as the number of pregnant females divided by the total number of inseminated females. Calving-first AI interval (days) is the calving to first service interval. Calving-calving interval (days) is the interval between the first and the second calving.

### Tissue sampling

Subcutaneous adipose tissue biopsies were carried out on the same animals at 4, 8, 20 and 44 wk. pp. (Experiment 1) or at − 4, 1 and 16 wk. peripartum (Experiment 2). Cows were fasted for 12 h before surgery and anaesthesia was induced by intravenous (IV) injections of 12 to 14 mg of xylazine (Rompun, Bayer, Leverkusen, Germany). Subcutaneous fat was collected from the dewlap under the neck and immediately frozen in liquid nitrogen. Briefly, the area of each biopsy sampling point was shaved, washed and disinfected with 70% ethanol and iodine. Infiltration anaesthesia with 20 mg lidocaine (Lurocaïne, Vetoquinol, Lure, France) was applied. A 1.5 cm skin incision was made at the dewlap under the neck. After the sampling, the wounds were closed by a suture, treated with aluminum spray and monitored every day until healing. In the experiment 1, before SAT biopsy and ovarian follicles aspiration, ovarian ultrasonographic examinations were performed with a transrectal ultrasonographic examination using a linear probe (LV 513 6.0/8.0-MHz transducer; Mylab30; Esaote) to determine the stage of the estrous cycle. During the luteal phase, the aspiration of small follicles (3–5 mm) was performed during transvaginal ultrasound scanning of ovaries on the same animals at 8, 20 and 44 wk. pp. (Experiment 1). The follicular fluids from several small follicles were pooled for each cow (small follicles from both ovaries were aspirated), centrifuged (800×g, 5 min) and then red blood cells were discarded from the pellet using two layers of discontinuous Percoll gradient (40, 60% in modified McCoy 5A medium supplemented with 20 mM Hepes, penicillin (100 units [U]/mL), streptomycin (100 mg/L), L-glutamine (3 mM), 5 mg/L transferrin, and 20 mg/L selenium (Thermofisher Scientific, Villebon-sur-Yvette, France)) and centrifugation (800×g, 20 min). To remove the remaining of red blood cells, the 40% fraction was then treated with hemolytic medium (NH_4_Cl 10 mmol/L in Tris HCl at pH 7.5; Sigma, Isles d’Abeau, France), centrifuged (800×g, 5 min) and then purify again on the discontinuous Percoll gradient. After centrifugation, the pellet was washed with fresh medium (modified McCoy 5A). We checked the purity of the granulosa cells in the pellet after fixation of one aliquot of the cells with paraformaldehyde 4% and immunocytochemistry with the 3-beta (β)-hydroxysteroid dehydrogenase antibody (3β-HSD (A-1) antibody sc-515,120 from Santa Cruz Biotechnology, Heidelberg Germany, data not shown). The remaining of the pellet was frozen at − 80 °C until use. Tissue or cell samples (SAT and GC) were stored at − 80 °C until use.

Nine pools of large (> 7 mm), medium (> 5 and ≤ 7 mm) and small (3–5 mm) follicles were recovered from a local slaughterhouse to determine the transcript expression of RARRES2 and CMKLR1. Each pool of large and medium follicles was obtained from 5 animals where each pool of small follicles was from one animal.

### Plasma metabolites and hormones

Blood samples were taken from coccygeal vein into heparinized Vacutainers (Dutcher, Brumath, France) before diet distribution, once weekly from calving to 44 wk. pp. (Experiment 1) or once every 2 wk. from 4 wk. before calving until calving and weekly from calving to 16 wk. pp. (Experiment 2). For the measurement of plasma progesterone (experiment 2), blood samples were taken three times a week. Blood was immediately centrifuged (2000×*g* for 15 min at 4 °C) and the separated plasma was stored at − 20 °C until assay. Non-esterified fatty acids (NEFA) and glucose were determined by enzymatic colourimetry assays (Wako Chemicals, Neuss, Germany; Sigma Aldrich, Saint Quentin-Fallavier, France). The intra- and inter-assay coefficients of variation (CV) of both plasma NEFA and glucose measures were 6 and 7.8%, respectively. Plasma insulin was measured by RIA from 100 μL of undiluted plasma as previously described [25]. Plasma progesterone was measured with an ELISA assay [31] from 10 μL of undiluted plasma from dairy cows and the detection limit of the assay was 0.4 ng/mL. The intra- and inter-assay coefficients of variation for progesterone were less than 6 and 7% respectively. Plasma bovine RARRES2 concentrations were analysed in duplicate from 100 μL of undiluted plasma by using a specific bovine RARRES2 ELISA kit (E11C0104; Holzel Diagnostika, Köln, Germany), with intra- and inter-assay of 4.5 and 6.5%, respectively. The antibody used in the bovine RARRES2 ELISA kit is a polyclonal antibody from rabbit; immunogen is recombinant full-length bovine protein from “*E. coli*”. The standard condition was performed with recombinant full-length bovine protein from “*E. coli*”. The ELISA assay detect both proRARRES2 and RARRES2 according to the distributor. The sensitivity of this assay is 0.1 ng/mL. We also determined the linearity of this assay, for this we diluted bovine plasma 1: 2, 1: 5, 1: 10 and 1: 20 and we observed a % of linearity between 90 and 100%. All assays and ELISA were investigated in duplicate.

### RT-qPCR

Total RNA of SAT and GC of all animals was extracted by homogenization in the TRIzol reagent using an Ultra-turrax (Invitrogen by Life Technologies, Villebon sur Yvette, France) according to the manufacturer’s recommendations. Concentration and purity of isolated RNA were determined with a NanoDrop Spectrophotometer (Peqlab Biotechnologie, Erlangen, Germany). Furthermore, the integrity of RNA was checked on 1.25% agarose-formaldehyde gels. Equal amounts of RNA treated with DNaseI (Thermo Fisher Scientific, Saint Herblain, France) from each pool of GCs or SAT sample from one cow at different peripartum or lactation times or from pool of GCs from different sizes of follicles from several cows were used for reverse transcription. Complementary DNA was synthetized from 1 μg of total RNA using MMLV (Promega, Charbonnières-les-Bains, France) according to the manufacturer’s instructions. Briefly, the cDNA was generated by reverse transcription (RT) of total RNA in a 20 μL mixture comprising: 0.5 mM each deoxyribonucleotide triphosphate (dATP, dGTP, dCTP, dTTP), 4 μL of 5X RT buffer, 0.15 μg of oligodT, 20 U of ribonuclease inhibitor and 200 U MMLV (Moloney murine leukemia virus reverse transcriptase) for 1 h at 37 °C.

After the RT, cDNAs were diluted 1:5. The 20 μl reaction mixture for real-time PCR contained 10 μl iQ SYBR Green supermix (Bio-Rad, Marnes-la-Coquette, France), 0.25 μl of each primer (at a final concentration of 150 nM, Table 1), 4.5 μl of sterile water, and 5 μl of diluted cDNA template. Real-time PCR reactions were carried out on a CFX96 (Bio-Rad, Marnes-la-Coquette, France). The efficiency of each primers (Table 1) and standard curve for each gene were calculated from serial dilutions of the corresponding cDNA fragment obtained as a template. The samples were duplicated on the same plate according to the following procedure: after an incubation of 2 min at 50 °C and a denaturation step of 10 min at 95 °C, samples were subjected to 40 cycles (30 s at 95 °C, 30 s at 60 °C, 30 s at 72 °C). All the amplified cDNA fragments were checked by automatically sequencing (Cogenics, Meylan, France). To evaluate the risk of amplification from genomic DNA potentially present in our total RNA preparations, all samples were amplified by PCR with each primer pair in the absence of reverse transcriptase. When no enzyme was present, no amplification product was detected (data not shown). The levels of mRNA expression were standardized to the geometric mean of three reference genes (UXT (Ubiquitously-expressed prefoldin-like chaperone), SDHA (Succinate dehydrogenase complex subunit A flavoprotein) and PPIA (Cyclophilin A)). Indeed, the geometric mean of multiple housekeeping genes has been reported as an accurate normalization factor [32]. The geometric mean of these three reference genes was stable in the different conditions studied. The relative amounts of gene transcripts (R) were calculated according to the equation: R = (E_gene_^−Ct gene^)/(geometric mean (E_UXT_^−Ct UXT^; E_SDHA_^−Ct SDHA^; E_PPIA_^−Ct PPIA^)), where E is the primer efficiency and Ct the cycle threshold.

**Table 1:** List of oligonucleotide primer sequences

Gene name	Forward 5′–3′	Reverse 5′–3′	Size (bp)	Accession number	PCR efficiency
UXT	CATTGAGCGACTCCAGGAAG	GGCCACATAGATCCGTGAAG	111	NM 001037471.2	2.01
SDHA	TATATGGCGCTGGCTGTCTC	CCTCTTCCCTCGCGGATTTC	168	XR 003031167.1	1.95
PPIA	GCATACAGGTCCTGGCATCT	TGTCCACAGTCAGCAATGGT	216	NM 178320	2.00
RARRES2	GAGGAGTTCCACAAGCATC	ACCTGAGTCTGTATGGGACA	265	XM 024990576.1	1.90
CMKLR1	CGGCCATGTGCAAGATCAGC	CAGGCTGAAGTTGTTAAAGC	259	XM 005218095.3	2.00

*UXT* Ubiquitously expressed transcript protein, *SDHA* Succinate dehydrogenase complex, subunit A, *PPIA* Peptidylpropyl isomerase A, *RARRES2* Retinoic acid receptor responder 2, *CMKLR1* Chemokine like receptor 1

### Statistical analysis

All statistical analyses were performed with SAS software (version 9.3; SAS Institute, Cary, NC). If not specified, data were analysed using the MIXED procedure for linear mixed models and we included “cow” as a variable. A repeated effect of time (week peripartum or pp) within animals was tested. The residuals from the observations generated from the mixed models were tested for normal distribution. The obtained values were normal distributed. The statistical model included the fixed effect of week for both experiment and diet, week, diet × week interaction as previously described [25]. The CORR procedure was used to calculate and measure the strength and direction of associations that exist between variables measured. The area under the curve (AUC) of plasma RARRES2 concentrations was performed as the sum of linear AUC that was calculated according to the following formula: AUC = x (t1) + y (t2) × 2/(t2 – t1) with x and y as a value of plasma RARRES2 concentration at 2 times (t1 or t2). Pregnancy rate after AI1 and the percentage of follicular waves were compared between the two groups of animals (LE and HE) using a Chisquare test with the FREQ procedure of SAS. For quantitative reproductive parameters (Commencement of luteal activity, cycle length, number of follicles, and calving intervals means were compared with a unpaired (HE vs LE) t-test (StatView version 4.57, Abacus Concepts, Berkeley, CA). Results are represented as least squares means (LSM) ± SEM and differences with *P* ≤ 0.05 were considered significant.

## Results

### Zootechnical parameters, plasma metabolites and RARRES2 levels in dairy cows from 4 wk. pp. to the late lactation period (44 wk. pp): Experiment 1

**Fig. 1:**
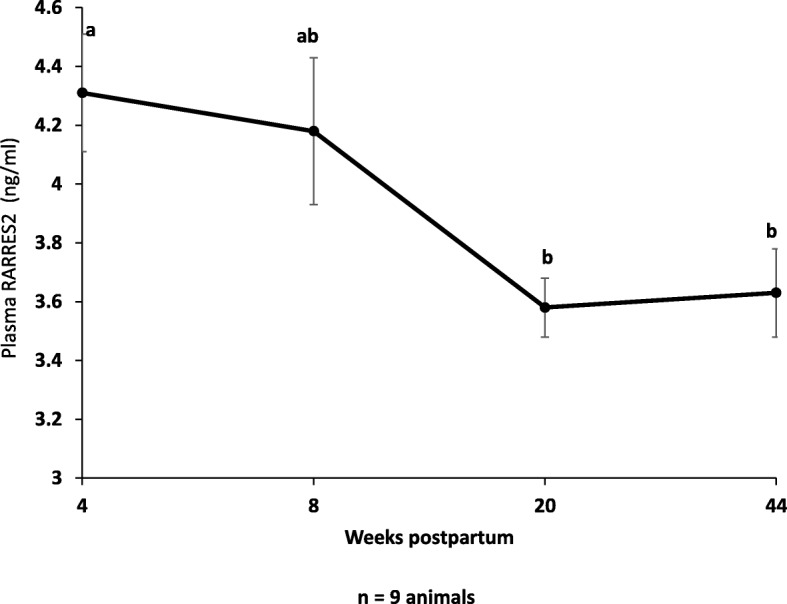
Plasma RARRES2 profile determined by ELISA from 4 wk. pp. to late lactation period (44 wk. pp) in lactating cows. Analysis of plasma RARRES2 concentrations at 4, 8, 22 and 44 wk. pp. of lactating cows was performed by ELISA (A, n = 9). Results are represented as LSM ± SEM and different letters indicate significant differences at *P* < 0.05

After calving, animals progressively increased the live body weight (*P* < 0.0001) and the variation of empty body weight (*P* < 0.0001) until 44 wk. pp. while they decreased their back fat thickness (*P* = 0.007). Animals also increased energy balance (*P* = 0.001) over time. In parallel, we observed a significant decrease in milk production at the late lactation period (*P* < 0.0001) while no significant effect was observed on the dry matter intake (*P* = 0.09) (Table 2). As expected, plasma NEFA concentrations significantly decreased until the late lactation period (*P* < 0.0001) (Table 2). We also noted an increase of plasma glucose (*P* = 0.01) and insulin (*P* = 0.0005) concentrations (Table 2). Interestingly, plasma RARRES2 concentrations were higher at 4 wk. pp. as compared to 20 and 44 wk. pp. (*P* = 0.009) as determined by ELISA (Fig. 1). Plasma RARRES2 levels were negatively correlated with variation of empty body weight (r = − 0.38, *P* = 0.02) and positively correlated with milk yield (r = 0.51, *P* = 0.003) and plasma NEFA levels (r = 0.41, *P* = 0.01).

**Table 2:** Zootechnical parameters and plasma metabolites and RARRES2 concentrations from 4 wk. pp. to late lactation period (44wk postpartum) in dairy cows

		Wk pp			
	4	8	20	44	*P*-value
	LSM ± SEM	LSM ± SEM	LSM ± SEM	LSM ± SEM	
Live body weight (kg)	640.74 ± 21.31^a^	643.16 ± 23.07^a^	685.01 ± 21.15^a^	741.49 ± 25.21^b^	**< 0.0001**
Variation of empty body weight (kg/d)	−14.05 ± 2.82^a^	− 13.86 ± 2.57^a^	− 8.26 ± 2.04^a^	− 0.71 ± 2.58^b^	**< 0.0001**
Milk yield (kg/d)	39.20 ± 2.27^a^	36.12 ± 2.97^a^	32.21 ± 1.48^a^	20.78 ± 1.60^b^	**< 0.0001**
Dry matter intake (kg/d)	18.67 ± 1.56^a^	20.83 ± 0.76^a^	21.41 ± 1.05^a^	21.77 ± 0.69^b^	0.09
Energy balance (Mcal/d)	− 4.88 ± 1.72^a^	−1.76 ± 1.62^ab^	−0.97 ± 1.35^ab^	2.76 ± 0.92^b^	**0.001**
Back fat thickness (cm)	0.65 ± 0.15^a^	0.43 ± 0.12^ab^	0.34 ± 0.11^b^	0.45 ± 0.09^b^	**0.007**
Non esterified fatty acid (mmol/L)	1103.98 ± 158.30^a^	816.58 ± 137.67^ab^	509.33 ± 75.00^b^	185.83 ± 29.48^c^	**< 0.0001**
Glucose (mmol/L)	7.71 ± 0.39^a^	8.17 ± 0.50^ab^	9.22 ± 0.44^b^	9.51 ± 0.57^b^	**0.01**
Insulin (ng/mL)	0.24 ± 0.06^a^	0.33 ± 0.05^ab^	0.49 ± 0.06^bc^	0.55 ± 0.02^c^	**0.0005**

Results are presented as LSM. *P*-values of the effects of week was considered significant if *P* < 0.05

### Expression of RARRES2 and CMKLR1 mRNA in SAT and GC of dairy cows from 4 wk. pp. to the late lactation period (44 wk. pp): Experiment 1

**Fig. 2:**
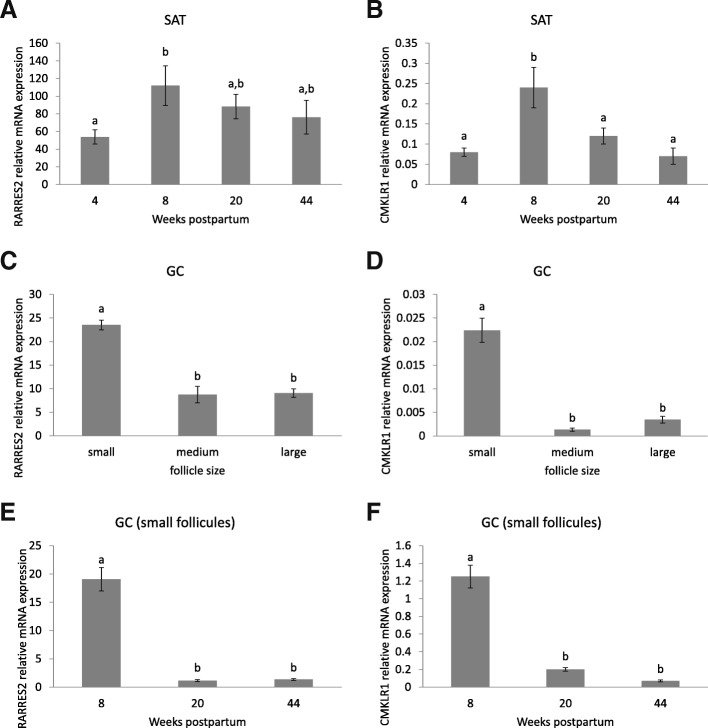
Expression of RARRES2 and CMKLR1 mRNA in SAT and GC in lactating cows. Analysis of RARRES2 (**a**) and CMKLR1 (**b**) mRNAs expression in SAT at 4, 8, 20 and 44 wk. pp. was performed by qRT-PCR (*n* = 9). We also measured the expression of RARRES2 (**c** and **e**) and CMKLR1 (**d** and **f**) mRNA in GC from different seize of follicles collected randomly in a slaughterhouse (**c** and **d**) and in GC from small follicle collected on the same animals as for SAT at 4, 8, 20 and 44 wk. pp. in lactating cows (*n* = 9). Results are represented as LSM ± SEM and different letters indicate significant differences at *P* < 0.05

In SAT, the expression of RARRES2 mRNA increased from 4 to 8 wk. pp. (*P* < 0.05) and then was unchanged from 8 to 20 or 44 wk. pp. (Fig. 2a). CMKLR1 mRNA expression also increased from 4 to 8 wk. pp., then decreased from 8 to 20 wk. pp., and then remained stable from 20 wk. pp. to the late lactation period (Fig. 2b). We next investigated the expression of RARRES2 and CMKLR1 in GC from different size of follicles from ovaries collected in a local slaughterhouse. The expression levels of RARRES2 and CMKLR1 mRNA were higher in GC from small follicles than in medium and large follicles (Fig. 2c and d). So, in Experiment 1, we collected by aspiration without FSH stimulation, GC from small follicles at 8, 20 and 44 wk. pp. The transcript levels of RARRES2 and CMKLR1 significantly decreased from 8 to 20 wk. pp. and no difference was observed between 20 to 44 wk. pp. (Figs. 2e and f). The expression of RARRES2 mRNA in GC from small follicles was positively correlated with plasma RARRES2 levels from calving to the late lactation period (r = 0.52, *P* = 0.03) and its mRNA expression in SAT was positively correlated with plasma RARRES2 levels only at 4 wk. pp. (r = 0.90, *P* = 0.0003).

### Zootechnical parameters in dairy cows fed either with HE or LE diet during the peripartum period in primiparous cows: Experiment 2

Zootechnical parameters including live body weight, variation of empty body weight, milk yield, dry matter intake, energy balance and back fat thickness were measured from − 4 to 16 wk. peripartum in primiparous cows (Additional file 2: Figure S1). During peripartum period, all zootechnical parameters were affected by week (*P* < 0.05). Live body weight and variation of empty body weight decreased from − 4 (or 1) to 8 wk. peripartum. In parallel, we observed a variation of milk yield over time (*P* = 0.005) but no effect between the two groups of cows (*P* = 0.34, Additional file 2: Figure S1E). Dry matter intake, and the energy balance increased and the back fat thickness decreased with time for every animal. Dry matter intake, the energy balance and the variation of empty body weight were affected by the diet during peripartum period (*P* < 0.05) while no effect of diet was observed on the other parameters. The effect of diet was marked by higher values in HE animals than in LE animals. Live body weight, the milk yield and the back fat thickness were also affected by the interaction of diet and week during the peripartum period (Additional file 2: Figure S1).

### Plasma metabolites and RARRES2 levels in dairy cows fed either with HE or LE diet during the peripartum period of primiparous cows: Experiment 2

**Fig. 3:**
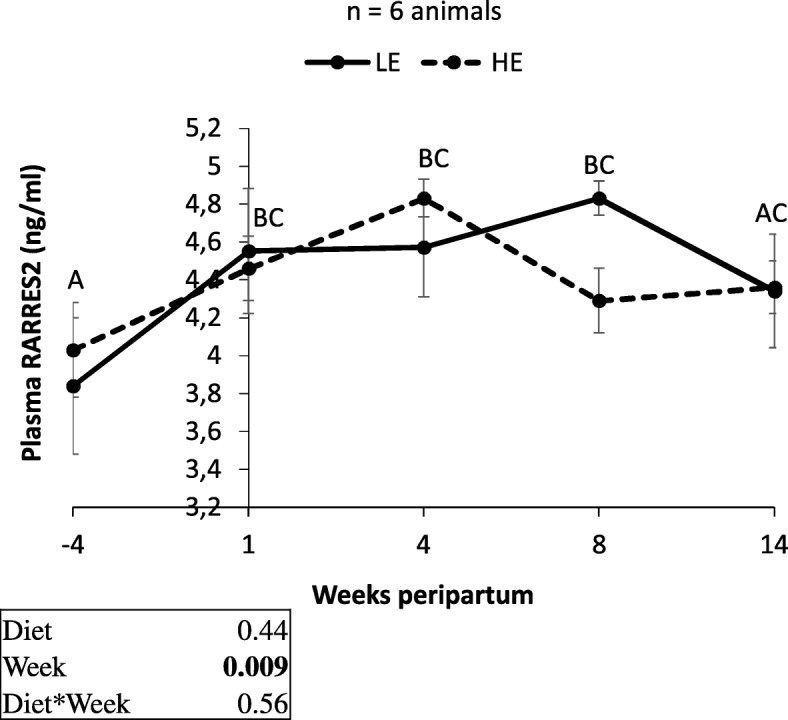
Plasma RARRES2 profile in primiparous dairy cow fed either with high-energy (HE) or low-energy (LE) diets. Analysis of plasma RARRES2 concentrations at −4, 1, 4, 8 and 16 wk. peripartum of dairy cows fed either with high-energy (HE, *n* = 6 or 8) or low-energy (LE, n = 6 or 8) was performed by ELISA. Results are represented as LSM ± SEM. When we observed a significant effect of the week without effect of the diet we represented difference with capital letter while when we observed a significant effect of the week and of the diet we represented separately the differences with low case letters for LE (bold) and HE (pale) cows. P-values were considered significant if *P* < 0.05

Plasma NEFA, glucose, insulin and RARRES2 concentrations were assessed from − 4 to 14 wk. peripartum (Additional file 2: Figure S2, Fig. 3). Plasma NEFA and RARRES2 concentrations were affected by the week during the peripartum period whereas plasma glucose concentrations were only affected by the diet (*P* = 0.04). Plasma NEFA concentrations increased from − 4 to 1 wk. peripartum and decreased from 4 to 14 wk. pp. (Additional file 2: Figure S2A). Plasma RARRES2 concentration increased from − 4 to 4 wk. peripartum whereas it was not affected by the diet (Fig. 3). An effect of diet was noted only on plasma glucose. In addition, we found that plasma RARRES2 concentration was negatively correlated with live body weight (r = − 0.23, *P* = 0.004), variation of empty body weight (r = − 0.47, *P* < 0.0001), energy balance (r = − 0.23, *P* = 0.008) and plasma glucose concentration (r = − 0.15, *P* = 0.05) and positively correlated with plasma NEFA concentrations (r = 0.30, *P* = 0.0001) during the peripartum period (Table 3).

**Table 3:** Pearson correlation coefficient (r) calculated between plasma RARRES2 as determined by ELISA and zootechnical parameters or plasma metabolite hormones from − 4 to 16 wk. peripartum in primiparous Holstein dairy cows fed either with a high or low energy diet

		LBW^1^	VEBW^2^	MY^3^	DMI^4^	EB^5^	BFT^6^	NEFA^7^	Glucose	Insulin
Plasma RARRES2	r	**− 0.23**	**−0.47**	0.13	−0.11	**− 0.23**	−0.16	**0.30**	**−0.15**	− 0.14
	*P*	**0.004**	**< 0.0001**	0.14	0.22	**0.008**	0.20	**0.0001**	**0.05**	0.12

The correlation was noted “r” and P-value (*P*) was considered significant if *P* < 0.05 (bold). ^1^Live body weight, ^2^Variation of empty body weight, ^3^Milk yield, ^4^Dry matter intake, ^5^Energy balance, ^6^Back fat thickness, ^7^Non esterified fatty acids

### Expression of RARRES2 and CMKLR1 mRNA in SAT of dairy cows fed either a HE or LE diet during the peripartum period of primiparous cows: Experiment 2

**Fig. 4:**
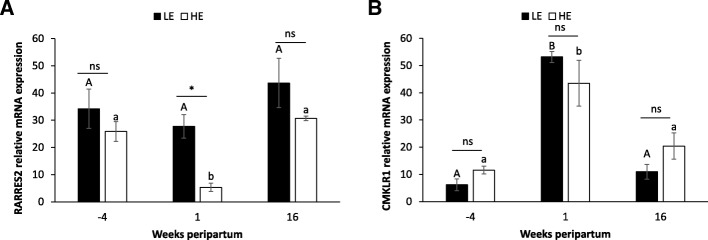
Expression of RARRES2 and CMKLR1 mRNA in SAT in primiparous dairy cow fed either with high-energy (HE) or low-energy (LE) diets. Analysis of RARRES2 (**a**) and CMKLR1 (**b**) mRNAs expression in SAT at − 4, 1 and 16 wk. peripartum of dairy cows fed either with high-energy (HE, *n* = 8) or low-energy (LE, *n* = 8) was performed by qRT-PCR. Results are represented as LSM ± SEM. When we observed a significant effect of the week without effect of the diet we represented difference with capital letter while when we observed a significant effect of the week and of the diet we represented separately the differences with low case letters for LE (bold) and HE (pale) cows. *P*-values were considered significant if *P* < 0.05. ns = no significant

The expression of RARRES2 mRNA was stable from − 4 to 16 wk. peripartum in LE animals while its expression significantly decreased from − 4 to 1 wk. peripartum and significantly increased from 1 to 16 wk. pp. in HE animals (Fig. 4a). The CMKLR1 mRNA expression significantly increased from − 4 to 1 wk. peripartum and significantly decreased from 1 to 16 wk. pp. in both HE and LE animals (Fig. 4b). We only found a significant lower expression of RARRES2 mRNA in HE as compared to LE animals at 1 wk. pp. (Fig. 4a).

### Reproductive parameters in dairy cows fed either with HE or LE diet and association with plasma RARRES2: Experiment 2

As described in the materials and methods, we determined in HE and LE animals (experiment 2) the cycle length, the number of follicular waves, the number of follicles (small, medium and large), the calving first artificial insemination and calving-calving intervals and the success rate at 35 and 90 days after artificial insemination. As shown in Table 4, we observed a significant difference between HE and LE animals only for the calving-first AI interval. Indeed, we found that LE animals had a longer calving-first AI interval than HE animals (*P* = 0.008). Interestingly, when we combined data from LE and HE animals, we observed a significant negative correlation between the number of small follicles and the AUC of plasma RARRES2 concentrations (r = − 0.56, *P* = 0.05, Table 5). In addition, if this correlation was analysed separately in LE and HE, it was significant only in HE animals (r = − 0.82, *P* = 0.04) and not in LE animals (r = − 0.52, *P* = 0.32).

**Table 4:** Reproductive parameters of cows fed either with high (HE) or low (LE) energy diet

	HE (*n* = 8)	LE (*n* = 8)	*P*-value
Commencement of luteal activity (days)	24.86 ± 4.07	31.88 ± 4.39	0.27
Cycle length (days)	33.13 ± 6.12	29.13 ± 3.91	0.59
Follicular waves (0)	12.5% (1:8)	25% (2/8)	0.60
Follicular waves (1)	0% (0/8)	25% (2/8)	0.18
Follicular waves (2)	12.5% (1:8)	12.5% (1:8)	1
Follicular waves (3)	50% (4/8)	0% (0/8)	0.07
Follicular waves (≥4)	25% (2/8)	37.5% (3/8)	0.70
Number of small follicles	44.13 ± 13.57	24.75 ± 8.57	0.25
Number of medium follicles	9.63 ± 2.94	5.38 ± 2.01	0.25
Number of large follicles	6.38 ± 1.21	3.75 ± 1.16	0.12
Calving-first AI interval (days)	**63.4 ± 3.97**	**94.5 ± 5.5**	**0.008**
Calving-calving interval (days)	367.8 ± 16.64	372.5 ± 0.5	0.87
Success rate 35 days after AI	37.5% (3/8)	50% (4/8)	0.75
Success rate 90 days after AI	37.5% (3/8)	50% (4/8)	0.75

**Table 5:** Pearson correlation (r) between AUC of plasma RARRES2 concentration and reproductive parameters including HE and LE animals

		AUC RARRES2
Commencement of luteal activity (days)	r	0.08
	*P*	0.83
Cycle length (days)	r	−0.26
	*P*	0.43
Number of follicular waves	r	−0.47
	*P*	0.12
**Number of small follicles**	**r**	**−0.56**
	***P***	**0.05**
Number of medium follicles	r	−0.34
	*P*	0.29
Number of large follicles	r	−0.40
	*P*	0.20
Calving-first AI interval (days)	r	0.10
	*P*	0.88
Calving-calving interval (days)	r	−0.69
	*P*	0.24
Success rate 35 days after AI	r	−0.13
	*P*	0.70
Success rate 90 days after AI	r	−0.13
	*P*	0.70

The correlation was noted “r” and P-value (*P*) was considered significant if *P* < 0.05 (bold). *AUC* Area under the plasma RARRES2

## Discussion

In the present study, we showed for the first time that the plasma RARRES2 concentration, as determined by ELISA, is significantly higher in early lactation when fat and energy mobilization is important as compared to the late lactation period when there is a higher energy balance. Furthermore, similar variations were observed for the expression level of RARRES2 and CMKLR1 mRNA in SAT and GC from small follicles. We also showed that in our experimental condition the energy content of the diet did not affect plasma RARRES2 concentration, and adipose CMKLR1 mRNA expression in peripartum whereas it significantly modified the expression level of RARRES2 mRNA in adipose tissue. Indeed, at 1 wk. pp., the expression levels of RARRES2 were significantly lower in SAT from HE animals as compared to LE animals. In addition, we observed that the area under the curve for plasma RARRES2 was significantly negatively associated with the number of small follicles in HE animals. However, this result was not observed in LE animals.

In dairy cows, the onset of lactation increases the total energy requirements for milk, exceeding the available energy from feed intake [33]. Herein, in our two experiments (1 and 2), we confirmed that during early lactation the shortage in energy resulting from the imbalance between energy input and energy output lead to a NEB and weight loss [34]. This NEB is associated with an elevated concentration of serum NEFA reflecting intensive fat reserve mobilization [35]. In good agreement with previous studies from the literature [36], in our experiment 1 we also found that at the beginning of lactation (4 wk. pp) plasma insulin and glucose levels are decreased compared to the mid (20 wk. pp) and late lactation periods (44 wk. pp). The switch towards lipolysis at the expense of lipogenesis increased circulating concentrations of NEFA. These metabolic variations are coordinated by changes in the plasma concentration of key hormones, including adipokines [37, 38]. Previous and recent data from the literature revealed that adipose tissue metabolism in lactating dairy cattle is essential in the successful establishment and support of lactation efficiency [39, 40]. The RARRES2/CMKLR1 system is considered to be a potential actor underlying the regulation of glucose and fat metabolism linked to obesity in humans and mice [9, 41, 42].

In our study, we showed for the first time that plasma levels of RARRES2 are high during early lactation, then decrease after 20 wk. of lactation. We determined plasma RARRES2 levels by using a specific bovine ELISA kit. We obtained values for plasma RARRES2 concentration ranging between 3.58 and 4.83 ng/ml. These values are low as compared to those described in humans (100–200 ng/ml) [43, 44] suggesting that plasma RARRES2 concentration is dependent on the species. The antibody used in the bovine RARRES2 ELISA kit is a polyclonal antibody from rabbit; immunogen is recombinant full-length bovine protein from “*E. coli*”. The standard condition was performed with recombinant full-length bovine protein from “*E. coli*”. We don’t know if this protein has the same functionality as the native bovine protein (post-translational modifications, e.g. glycosylation and folding). However, with this ELISA kit, we found that the concentration of plasma RARRES2 was positively correlated with those of NEFAs, indicating a potential involvement in fatty acid metabolism and more precisely in lipolysis. These data are in good agreement with those described by Suzuki et al. [45, 46] showing that administration of RARRES2 in sheep increases plasma NEFA levels. Furthermore, a recent study from Fu et al. [47] showed that RARRES2 is able to induce significant lipolytic metabolism in bovine intramuscular mature adipocytes. In our study, we did not analyse the intramuscular adipose cells but rather the SAT. However, we can speculate that RARRES2 could have the same lipolytic role in the mature adipocytes located in different places. A lipolytic effect of RARRES2 has also been described in differentiated 3 T3-L1 adipocytes [11]. In bovine species, the expression of RARRES2 mRNA is upregulated during adipogenesis [3, 48]. Here, in the experiment 2, in the animals fed with the high energy diet we found lower mRNA expression of RARRES2 during early lactation (1 wk. pp) than in prepartum (− 4 wk. peripartum) or at 20 wk. pp. and opposite expression patterns were obtained for CMKLR1 in SAT. This opposite pattern regulation between RARRES2 and CMKLR1 was also observed in LE animals. In addition, the adipose tissue physiology is mediated by the dynamic of multiple adipokines. Suzuki et al. [48] showed that TNF-α and adiponectin regulated in opposite the expression of RARRES2 and CMKLR1. So we can speculate that the effect on RARRES2 and CMKLR1 expression is not direct or that RARRES2 may induce a decrease in CMKLR1 expression. Also, as already shown for different receptors such as insulin receptors, we can hypothesize that high or low expression of the ligand may down or up regulate its receptor [49]. Taken together, our data suggest that modifications of plasma RARRES2 and expression of RARRES2 mRNA in SAT observed immediately after calving may contribute to lipid mobilization during early lactation in dairy cows but supplemental experiments are needed to confirm this hypothesis. It is well known that RARRES2 augments insulin-stimulated glucose uptake, insulin signalling and improves insulin sensitivity in murine adipocytes [50]. However, our data indicated no association between concentrations of serum glucose or insulin.

The adaptations of increased lipolysis in adipose tissue during lactation can be attenuated by dietary energy intake in different mammalian species [25, 51–53]. We recently demonstrated that circulating levels of leptin and adiponectin were lower and those of resistin were higher in cows fed with a LE diet [25]. In addition, Chamberland et al. [54] showed that decreasing levels of RARRES2 in serum due to starvation are mediated through leptin in human. Based on these results, it was expected that circulating RARRES2 would respond appropriately to changes in energy intake in cows. Even though the LE diet impaired milk production, negative energy balance, dry matter intake and back fat thickness; no variation in plasma RARRES2 levels was observed as compared to animals fed with a HE diet. By contrast, we found that during early lactation (1 wk. pp) the expression of RARRES2 mRNA was lower in SAT of animals fed with a HE diet than animals fed with a LE diet and no variation in the expression of CMKLR1 mRNA was observed. We can hypothesize that a more drastic diet, with very low energy level, would be necessary to achieve the regulation of plasma RARRES2 level and consequently play retro-control loops in its local synthesis in the adipose tissue and liver. Indeed in rodents and humans, RARRES2 is produced by hepatocytes. However, the relative contribution of the liver versus adipose tissue to the RARRES2 blood concentrations remains unclear. Further experiments are necessary to demonstrate our hypothesis. In addition, our results are from one specific location of SAT and need to be confirmed in other locations of SAT including tailhead fat and other adipose tissues such omental, mesenteric or retroperitoneal.

Concomitantly, adipose tissue was described as a key player in supporting the overall efficiency of reproduction performance in multiple species, especially through the synthesis and release of adipokines. In rat, treatment with dihydrotestosterone induces polycystic ovarian syndrome associated with metabolic disorders and elevated ovarian RARRES2 levels [14]. In humans, levels of RARRES2 mRNA increased in maternal adipose tissue during normal gestation possibly contributing to the increase of maternal circulating RARRES2 concentrations that are associated with maternal body mass index and insulin resistance [55]. In bovine, we recently detected RARRES2 and CMKLR1 mRNA in bovine ovarian follicles [21]. In addition, in cultured bovine granulosa cells, RARRES2 decreases steroidogenesis and cholesterol synthesis [21]. Furthermore, we showed that the addition of RARRES2 to bovine complex cumulus-oocyte maturation medium arrested most of the oocytes at the germinal vesicle stage [21]. However, nothing was described concerning the expression of the RARRES2/CMKLR1 system in GC during a period of drastic metabolic modification such as lactation in bovine. Here, we found that the expression of RARRES2 and CMKLR1 mRNA were decreased after 20 wk. pp. in GC from small follicles and this pattern was correlated with plasma RARRES2 levels. This raised the hypothesis of the potential involvement of the RARRES2/CMKLR1 system in ovarian regulation during lactation and it may be important to study their molecular mechanisms at different stages of folliculogenesis. However, if its effect on granulosa cells is similar to those described in vitro we can speculate that high levels of RARRES2 may reduce progesterone production and consequently may affect folliculogenesis. Plasma RARRES2 could modulate granulosa cell function through CMKLR1. In vitro we showed that RARRES2 inhibited granulosa cell steroidogenesis and the MAPK ERK1/2 signaling pathways through CMKLR1 by using a blocking antibody [21]. RARRES2 has been demonstrated to regulate various signalling pathways [7].Two other receptors, called GPR1 (G Protein-Coupled Receptor 1) and CCRL2 (C-C chemokine receptor-like 2), are shown to bind RARRES2 in rodents. Even if the biological role and the intracellular signalling of these RARRES2 receptors are poorly understood, it will be interesting to investigate their pattern expressions in peripheral and reproductive tissues to better understand the complexity of the RARRES2 system that links metabolic and fertility efficiency in dairy cows. In our present data, we showed a significant negative correlation between the number of small follicles and the AUC of plasma RARRES2 concentrations and this association was more significant in HE animals. A significant positive association between the number of small follicles and the AUC of adipokine called resistin has already been described in dairy cows [25]. For RARRES2 our results need to be further analysed. Studies with a larger number of animals would be necessary to permit the elucidation and the functionality of the relation. In addition, we need to associate plasma RARRES2 or tissue RARRES2 expression with more molecular reproductive parameters. In this context, further investigations are in progress to evaluate the network related to RARRES2 in SAT and reproductive cells (GC and oocyte) by RNA sequencing.

## Conclusions

In conclusion, we showed the fat and energy moblization during lactation influences circulating RARRES2 levels as well as the expression of RARRES2 and CMKLR1 in SAT and similarly in ovarian cells in Holstein dairy cows. In SAT, the energy level of the diet regulates the expression of RARRES2. Furthermore, we observed that the area under the curve of plasma RARRES2 is negatively associated to the number of small follicles in HE animals suggesting that plasma or ovarian RARRES2 could be a signal involved in the reproductive functions in Holstein dairy cows.

## Additional files

Additional file 1: **Table S1A.** Composition and energy content of the HE and LE diets (% of DM). **Table S1B.** Chemical composition and nutritional value of feeds. (DOCX 14 kb)

Additional file 2: **Figure S1.** Zootechnical parameters in dairy cows fed either with HE or LE diet during the peripartum period in primiparous cows. Live body weight (A), dry matter intake (B), variation of empty body weight (C), energy balance (D), milk yield (E) and back fat thickness (F) of primiparous dairy cows fed either a HE diet or a LE diet was evaluated between − 4 and 16 wk. peripartum. Results are presented as LSM ± SEM. *P*-values of diet, week and the interaction between diet and week effect are presented. When we observed a significant effect of the week without effect of the diet we represented difference with capital letter while when we observed a significant effect of the week and of the diet we represented separately the differences with low case letters for LE (bold) and HE (pale) cows. P-values were considered significant if *P* < 0.05. **Figure S2.** Plasma metabolites levels in dairy cows fed either with HE or LE diet during the peripartum period of primiparous cows. Plasma concentrations of non esterified fatty acids (A), glucose (B) and insulin (C) of primiparous dairy cows fed either a HE diet or a LE diet was evaluated at − 4, 1, 4, 8 and 14 wk. peripartum**.** Blood samples were collected weekly before the morning diet distribution. Results are presented as LSM ± SEM. P-values of diet, week and the interaction between diet and week effect are presented. When we observed a significant effect of the week without effect of the diet we represented difference with capital letter while when we observed a significant effect of the week and of the diet we represented separately the differences with low case letters for LE (bold) and HE (pale) cows. *P*-values were considered significant if *P* < 0.05. (PDF 59 kb)
