# Supplementary material for: Possible involvement of the RARRES2/CMKLR1-system in metabolic and reproductive parameters in Holstein dairy cows
Source: Reprod Biol Endocrinol. 2019 Feb 18;17:25. doi: 10.1186/s12958-019-0467-x (PMC6380063; doi:10.1186/s12958-019-0467-x)
Supplement: Supplementary file 1 — Table S1A. Composition and energy content of the HE and LE diets (% of DM). Table S1B. Chemical composition and nutritional value of feeds. (DOCX 14 kb) [file 12958_2019_467_MOESM1_ESM.docx]

**Supplemental table 1A.** Composition and energy content of the HE and LE diets (% of DM).

|  |  | HE diet | |  | LE diet | |
| --- | --- | --- | --- | --- | --- | --- |
|  |  | Feed (% DM) | Energy Mcal/kg |  | Feed (% DM) | Energy Mcal/kg |
| Corn silage |  | 61.2 | 30.17 |  | 50.4 | 24.85 |
| Lucerne Hay |  | 10.1 | 9.03 |  | 10.2 | 9.12 |
| Grass silage |  | 0 | 0 |  | 24 | 14.97 |
| Energy Concentrate |  | 17.2 | 27.70 |  | 8.5 | 13.69 |
| Protein Concentrate |  | 10.7 | 17.33 |  | 6 | 9.72 |
| Ca Carbonate |  | 0.5 |  |  | 0.4 |  |
| Minerals CMV5/23 |  | 0.3 |  |  | 0.5 |  |
| Total |  | 100 | 84.23 |  | 100 | 72.35 |

**Supplemental table 1B**: Chemical composition and nutritional value of feeds

|  | Corn silage | Grass silage | Lucerne hay | Energy Concentrate | Protein Concentrate | Ca Carbonate | Minerals |
| --- | --- | --- | --- | --- | --- | --- | --- |
| DM, % | 31.6 | 45.2 | 86.8 | 88 | 90 |  |  |
| CP, g/kg DM | 71.6 | 137.3 | 131.3 | 19 | 53 |  |  |
| Cellulose, g/kg DM | 201.9 | 299.5 | 380.8 | 5 | 7 |  |  |
| Starch, g/kg DM | 292.3 |  |  | 327 | 55 |  |  |
| Energy, Mcal/kg DM | 1.56 | 1.38 | 1.03 | 1.83 | 1.80 |  |  |
| PDIN^1^, g/kg DM | 44.0 | 84.5 | 85.7 | 136 | 366 |  |  |
| PDIE^2^, g/kg DM | 66.7 | 70.7 | 79.8 | 136 | 244 |  |  |
| P, g/kg DM | 1.9 |  |  | 3.7 | 6.1 |  | 70 |
| Ca, g/kg DM | 2.0 |  |  | 2.5 | 2.3 | 350 | 220 |

^1^PDIN = protein digested in the small intestine supplied by rumen-undegraded dietary protein and by microbial protein from rumen-degraded dietary nitrogen (“Protéines Digestibles dans l’Intestin permises par l’azote” in French) (Jarrige, 1989), ^2^PDIE = protein digested in the small intestine supplied by rumen-undegraded dietary protein and by microbial protein from rumen-fermented organic matter (“Protéines Digestibles dans l’Intestin permises par l’Energie” in French) (Jarrige, 1989).
