# Supplementary figures and images for: Possible involvement of the RARRES2/CMKLR1-system in metabolic and reproductive parameters in Holstein dairy cows
Source: Reprod Biol Endocrinol. 2019 Feb 18;17:25. doi: 10.1186/s12958-019-0467-x (PMC6380063; doi:10.1186/s12958-019-0467-x)

A.

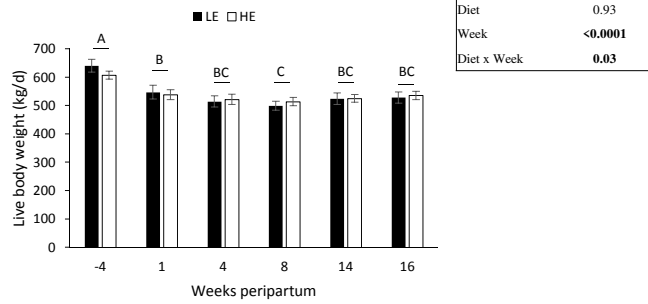

B.

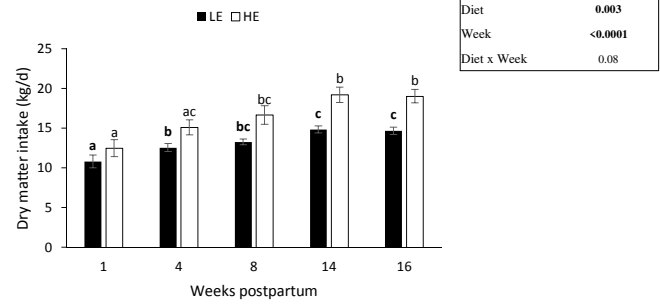

C.

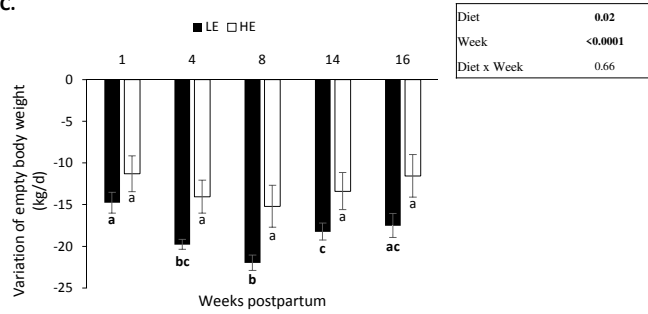

D.

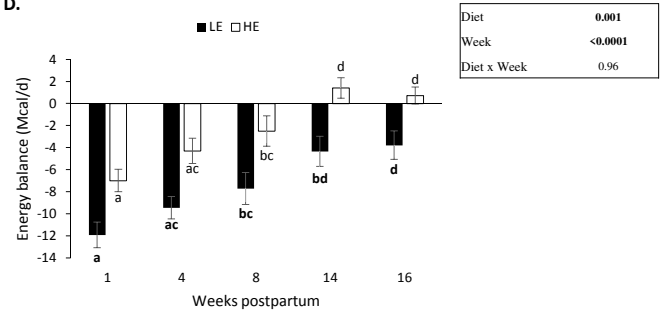

E.

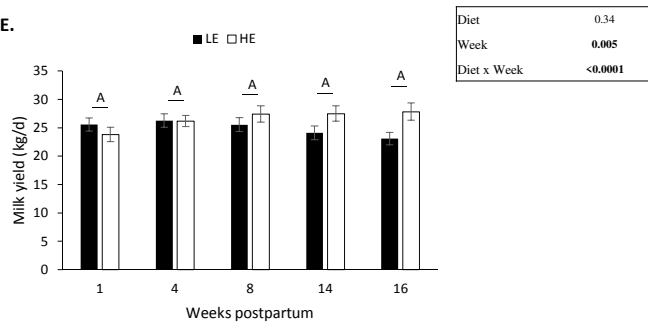

F.

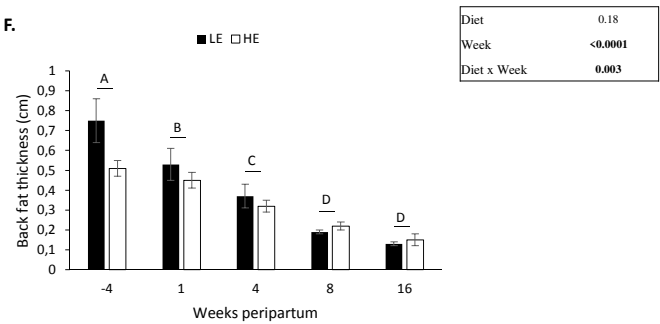

Supplemental figure 1

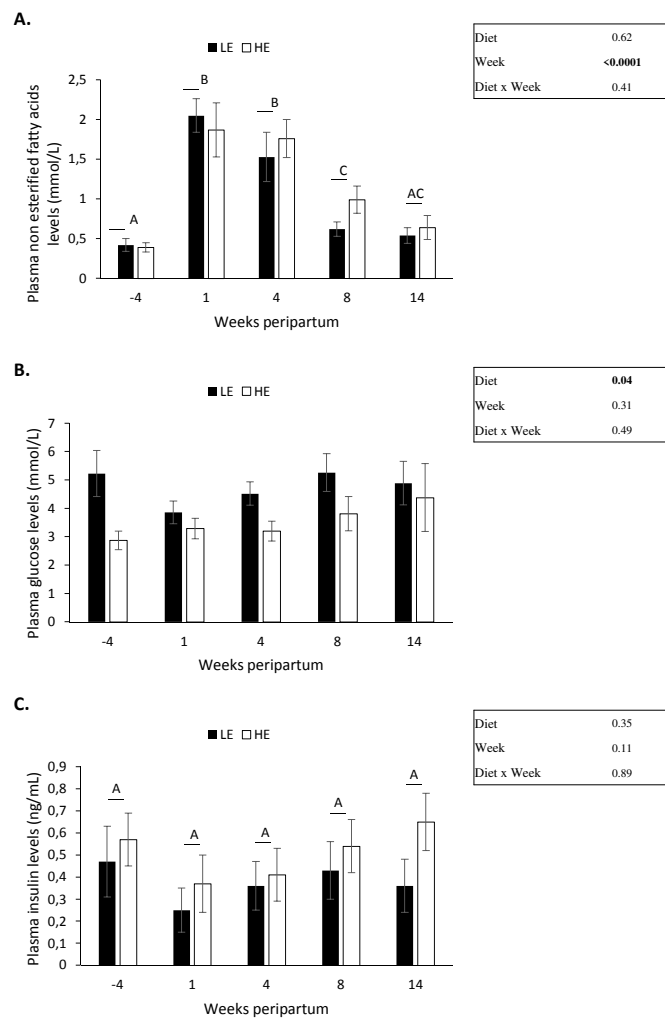

Supplemental figure 2

Supplement: Supplementary file 2 — Figure S1. Zootechnical parameters in dairy cows fed either with HE or LE diet during the peripartum period in primiparous cows. Live body weight (A), dry matter intake (B), variation of empty body weight (C), energy balance (D), milk yield (E) and back fat thickness (F) of primiparous dairy cows fed either a HE diet or a LE diet was evaluated between − 4 and 16 wk. peripartum. Results are presented as LSM ± SEM. P-values of diet, week and the interaction between diet and week effect are presented. When we observed a significant effect of the week without effect of the diet we represented difference with capital letter while when we observed a significant effect of the week and of the diet we represented separately the differences with low case letters for LE (bold) and HE (pale) cows. P-values were considered significant if P < 0.05. Figure S2. Plasma metabolites levels in dairy cows fed either with HE or LE diet during the peripartum period of primiparous cows. Plasma concentrations of non esterified fatty acids (A), glucose (B) and insulin (C) of primiparous dairy cows fed either a HE diet or a LE diet was evaluated at − 4, 1, 4, 8 and 14 wk. peripartum. Blood samples were collected weekly before the morning diet distribution. Results are presented as LSM ± SEM. P-values of diet, week and the interaction between diet and week effect are presented. When we observed a significant effect of the week without effect of the diet we represented difference with capital letter while when we observed a significant effect of the week and of the diet we represented separately the differences with low case letters for LE (bold) and HE (pale) cows. P-values were considered significant if P < 0.05. (PDF 59 kb) [file 12958_2019_467_MOESM2_ESM.pdf]
